# Author Correction: Structural basis for prostaglandin and drug transport via SLCO2A1

**DOI:** 10.1038/s41467-026-76870-0

**Published:** 2026-08-17

**Authors:** Chitra Joshi, Justin C. Deme, Yoshinobu Nakamura, Wei-Tse Hsu, Jonathan D. Goult, Takafumi Kato, Joanne L. Parker, Philip C. Biggin, Susan M. Lea, Takeo Nakanishi, Simon Newstead

**Affiliations:** 1 Department of Biochemistry, University of Oxford, Oxford, UK; 2 Kavli Institute of Nanoscience Discovery, University of Oxford, Oxford, UK; 3 Center for Structural Biology, Center for Cancer Research, National Cancer Institute, National Institutes of Health, Frederick, MD USA; 4 Faculty of Pharmacy, Takasaki University of Health and Welfare, Takasaki, Gunma Japan; 5 Gunma University Graduate School of Medicine, Maebashi, Gunma Japan; 6 Structural Bioinformatics and Computational Biochemistry, University of Oxford, Oxford, UK; 7 Structural Biology, St Jude Children’s Research Hospital, Memphis, TN USA

**Keywords:** Cryoelectron microscopy, Permeation and transport, Inflammation, Membrane proteins

Correction to: *Nature Communications* 10.1038/s41467-026-70227-3, published online 20 March 2026

In the version of this article initially published, in the Fig. 5c plot and associated Source Data, values for K549A were incorrect; in the Fig. 6c plot and associated Source Data, values for G222R were incorrect; in the Fig. 3b Source Data file, the control condition was mistakenly labeled “0.01 µM” rather than “0 µM.” The figures and Source Data are now amended in the HTML and PDF versions of the article.

